# European Association for Palliative Care White Paper on spiritual care for people with neuro-oncological and neurodegenerative conditions: Integrative framework for practice, education, and research

**DOI:** 10.1017/S1478951526102302

**Published:** 2026-04-20

**Authors:** Piret Paal, Reinhard Grabenweger, Sarah Bublitz, Elisabeth Bumes, Benno Littger, Joni Haikonen, Marie-José Gijsberts, David J. Oliver, Simone Veronese, Alexander Kowski, Megan Best

**Affiliations:** 1 Department of Ethnology, Institute of Cultural Research, Tartu University, Tartu, Estonia; 2 Institute of Nursing Science and Practice, Paracelsus Medical University, Salzburg, Austria; 3 Institute of Palliative Care, Paracelsus Medical University, Salzburg, Austria; 4 Department of Neurology – NeuroOncology and Wilhelm Sander-NeuroOncology Unit, University Hospital Regensburg, Regensburg, Germany; 5 Specialised Chaplaincy in Neurology, Archdiocese of Munich and Freising, Munich, Germany; 6 End of Life Research Group, Ghent University, Ghent, Belgium; 7 End of Life Research Group, Vrije Universiteit Brussel, Brussels, Belgium; 8 Tizard Centre, University of Kent, Canterbury, UK; 9 Palliative Care, Fondazione FARO Onlus, Turin, Italy; 10 Department of Neurology with Experimental Neurology, Charité – Universitätsmedizin Berlin Campus Charité Mitte, Berlin, Germany; 11 Institute for Ethics and Society, The University of Notre Dame Australia – Sydney Campus Broadway, Sydney, Australia

**Keywords:** Palliative care, spiritual care, EAPC, neuro-oncology, neurodegenerative disease, White Paper

## Abstract

**Objectives:**

This White Paper by the European Association for Palliative Care addresses the imperative to integrate spiritual care into the support of individuals living with neuro-oncological and neurodegenerative conditions. These diseases present complex biomedical, social, psychological, and existential challenges that demand a whole-person approach to care. Various initiatives have progressed the understanding of spirituality as a dimension of well-being, yet the systemic delivery of spiritual care remains inconsistent and inequitable.

**Methods:**

This study adopts a narrative umbrella review approach. We provide a synthesized framework highlighting current knowledge and models of care, educational needs, and future priorities for research, while advocating for the formal integration of spiritual care into all stages of illness.

**Results:**

Our exploration highlights the importance of early integration of dynamic and multidimensional spiritual care for people with neuro-oncological or neurodegenerative diseases. The implementation of spiritual care in this context should address the unique challenges that arise with these diseases, such as changes in spiritual needs and in the ability to communicate spiritual needs across disease progression. Spiritual care should be carried out by the whole care team, offering regular spiritual screenings and referring care to specialists when needed, and it should be offered across all stages of care. Spiritual care should be culturally safe, offering multilingual access, and multi-faith chaplaincy services.

**Significance of results:**

Spiritual care is not a luxury or an optional extra; it is a fundamental aspect of palliative care. There is a need to implement spiritual care across all stages of care, taking into consideration the patient’s evolving needs. Sufficient time should be allocated to spiritual care education for social and healthcare professionals. More research is needed to develop validated screening tools and effective interventions.

## Introduction

Neuro-oncological and neurodegenerative diseases, including primary and metastatic brain tumors, amyotrophic lateral sclerosis (ALS), Parkinson’s disease, and various forms of dementia, cause progressive motor and non-motor decline involving neurocognitive deficits, including aphasia, affecting people’s ability to interact with others and express their needs. This can occur very early on at the time of diagnosis and/or at a time when patients are experiencing recurrent loss, which is inherent in the disease trajectory. Loss experienced in relation to disease encompasses spiritual, cognitive, social, psychological, and physical dimensions with subsequent disruption of identity, relationships, and life narrative (Paal et al. 2020). The anticipation of death frequently invokes spiritual questions related to meaning, belonging, connection, and legacy (Best et al. 2015). In people diagnosed with cancer, such suffering has been described as “an all-encompassing, dynamic, individual phenomenon characterized by the experience of alienation, helplessness, hopelessness and meaninglessness in the sufferer which is difficult for them to articulate. It is multi-dimensional and usually incorporates an undesirable, negative quality” (Best et al. 2015).

Spiritual care, when integrated into early diagnosis, ongoing care, and end-of-life support, can support continuity of self, uphold dignity, help reduce spiritual distress, and improve quality of life (Puchalski et al. 2014; Garon et al. 2025). Therefore, timely access to neuropalliative care (palliative care for patients with neuro-oncological and neurodegenerative conditions or disease) (Oliver et al. 2016) that incorporates spiritual care is essential (Pace et al. 2017; Solari et al. 2020; Kluger et al. 2023). Yet, spiritual aspects are rarely considered in clinical care provided by neurologists (Boersma et al. 2014; Garon et al. 2024a, 2025; Brandstötter et al. 2025). Even though neuropalliative medicine encompasses more than the mentioned conditions, the focus of this manuscript is on neuro-oncological and neurodegenerative conditions.

Palliative care has historically been focused on patients with cancer diagnoses, and most patients with progressive neurologic illness lack access to high-quality palliative care services (Bublitz et al. 2024b). A more holistic approach is needed that addresses spiritual needs to improve quality of life.

## Methodology

This is a narrative umbrella review. Based on the limited scientific research on spiritual care for adults with primary and metastatic brain tumors (Mehta et al. 2024; Grabenweger et al. 2025b), as well as people with ALS (Gonçalves et al. 2023, 2025), Parkinson’s disease (Seshadri et al. 2023; Çavuşoğlu and Avcı 2024; Garon et al. 2024b, 2025), and Alzheimer’s disease (Britt et al. 2022), this European Association for Palliative Care (EAPC) White Paper on spiritual care for people with neuro-oncological and neurodegenerative conditions presents a rationale for elevating spiritual care to a core element of comprehensive and culturally safe neuropalliative care practice, education, and research. The discussion points were derived by the authors from the literature, discussed thoroughly, and synthesized into findings and future directions.

## Results

The seven findings were: (1) Spirituality and Religion: Conceptual Foundations, (2) Spiritual Needs, (3) Spiritual Care: Specialists and Generalists, (4) Spiritual History-taking, Spiritual Screening and Spiritual Assessment, (5) Challenges in Communication, (6) Cultural Safety in Spiritual Care, (7) Spiritual Care Across Care Settings (Clinical Inpatient Care; Community Care, Specialist Spiritual Care Chaplaincy).

### Spirituality and religion: conceptual foundations

In 2011, the Spiritual Care Task Force at the EAPC modified the U.S. consensus definition of spirituality (Puchalski et al. 2009) to arrive at the following working definition: “Spirituality is the dynamic dimension of human life that relates to the way persons (individual and community) experience, express and/or seek meaning, purpose and transcendence, and the way they connect to the moment, to self, to others, to nature, to the significant and/or the sacred.” Spirituality is best conceptualized as a multidimensional construct consisting of the following interrelated domains:

1. *Existential challenges* (e.g. questions concerning identity, meaning, suffering and death, guilt and shame, reconciliation and forgiveness, freedom and responsibility, hope and despair, love and joy).
2. *Value-based considerations and attitudes* (what is most important for each person, such as relations to oneself, family, friends, work, things, nature, art and culture, ethics and morals, and life itself).
3. *Religious considerations and foundations* (faith, beliefs, and practices; the relationship with God or the ultimate) (Nolan et al. 2011).

Religion and spirituality are overlapping but distinct terms (Hill et al. 2000). Religion can be understood as “a system of beliefs and practices that unites adherents into a community with a shared vision for attaining union with, or the experience of, the divine or transcendent” (Koenig and Carey 2024). Spirituality, as defined above, may or may not be expressed through religious practices. As most people hardly use the term “spirituality” in everyday conversation, listening carefully to the patient’s vocabulary is of major importance to discern ways to communicate about spirituality in the health-care setting (Best et al. 2020). Individuals with neuro-oncological and neurodegenerative conditions may express and experience spirituality in diverse ways, e.g. through faith, relationships, creativity, inner experiences, and images or experiences in nature. All are valid, and patients’ beliefs should be respected even if not shared by the health-care provider. A flexible, respectful, and inclusive conceptual spiritual care model is required to ensure that narrow interpretations do not restrict expressions or support of patient spirituality.

### Spiritual needs

Research reveals that patients’ spiritual needs are numerous and broad in scope, and common in patients with life-threatening disease (Grant et al. 2004). They may fluctuate in response to clinical events (Best et al. 2024).

In slow progressive neurological disease, spiritual needs may evolve over time because the physical changes that unfold can directly affect how individuals experience, interpret, and express spirituality (Paal et al. 2020). As neurodegenerative and neuro-oncological processes gradually alter brain structure, patients may experience shifts in their values, religious behaviors, and spiritual perceptions according to which part of the brain is affected. These changes may also occur suddenly as a result of tumor resection or new brain lesions in certain functional brain areas (Ferguson et al. 2022).

In the early stages, individuals may seek coherence or spiritual grounding in the face of uncertainty. In a large multinational, multicenter cohort study, people with late-stage Parkinson’s disease identified family, partnerships, and spirituality as their main sources of meaning in life but reported significantly lower importance and satisfaction in these core areas compared to healthy participants (Bublitz et al. 2024a). People living with mild dementia have identified central existential and spiritual concerns such as self-confidence and self-worth, adaptability and capacity, security and loss, burden and enrichment of memory, and faith and meaning (Haufe et al. 2024). It has been observed that spiritual needs can change over the course of neuro-oncological diseases, are particularly high at initial diagnosis and during progression, and are often not adequately addressed (Nixon and Narayanasamy 2010; Cavers et al. 2012; Philip et al. 2020).

As functional decline progresses, needs related to maintenance of dignity, autonomy, and meaning may become more pronounced as individuals become dependent on others to provide basic personal care. Many individuals are confronted by concerns about legacy, relational reconciliation, or spiritual preparation for death (Paal et al. 2020).

Families and informal caregivers may also articulate spiritual needs, such as the need to find peace (Brandstötter et al. 2025). They also face spiritual burdens, including anticipatory grief, role changes, and moral distress that deserve structured support (Paal et al. 2020). Importantly, evidence suggests that spiritual care of the patient will also benefit the carer (O’Callaghan et al. 2020).

### Spiritual care: specialists and generalists

Spiritual care generally refers to any clinical activities that recognize and support patients’ spiritual well-being in some way. It can be provided in any patient setting, including inpatient, outpatient/home care, and residential care, but may differ in nature according to the context. While chaplains provide expert spiritual support (spiritual care “specialists”), all team members (spiritual care “generalists”) must be equipped to recognize and respond to spiritual concerns (Puchalski et al. 2009; Jones et al. 2021b). Referral to the chaplain should be considered when spiritual needs are high, when specific clinical events ensue, or when the team member is unable to provide the spiritual care that is required (Jones et al. 2021b). Depending on the organization of specialist spiritual care services, chaplains might be in the privileged position to accompany the persons across care settings, which ensures continuity in care (Best et al. 2025).

Attention to the clinician’s own spirituality is often identified as the first step of spiritual care training (Best et al. 2020). Considering one’s own spirituality not only improves ability to identify patients’ spiritual needs but also encourages personal growth and reduces the risk that clinicians’ own existential distractions negatively impact patient care (Jones 1999; Koenig 2014).

### Spiritual history-taking, spiritual screening, and spiritual assessment

The first step of providing spiritual care to patients involves understanding the nature of the patient’s spirituality and identifying spiritual needs. Spiritual discussions are listed in the literature as spiritual history-taking and spiritual screening, both of which constitute a form of spiritual care. This is because spiritual discussion itself can develop into a therapeutic intervention (Cobb 2001). Spiritual history taking refers to the baseline collection of information about the source of spiritual strength for the patient, spiritual or religious practices and/or communities, and the impact of these on health-care decision-making and management. Spiritual screening refers to a periodical inquiry about spiritual needs, which can fluctuate and change over time. Many tools are available that can be used to conduct both types of assessment (Best et al. 2020, 2024). Some existing tools have been adapted for patients with impaired cognition, e.g. the Diamond model has been adapted for people with early-stage dementia (Haufe et al. 2024). Short screening questions, such as Are you at peace (with God)? (Steinhauser et al. 2006) or What’s most important to you right now? How can we help? (Ross and McSherry 2021), are helpful in identifying any momentary spiritual distress (Taylor 2020). Nevertheless, there is a lack of validated spiritual screening tools in the context of neurocognitive impairments, including aphasia, which often manifests early in the course of neurodegenerative and neuro-oncological conditions, and which can also be easily applied by spiritual care generalists in everyday practice. While all health-care professionals should be able to take a spiritual history or conduct brief spiritual screening, a detailed spiritual assessment should be carried out by chaplains if available (Kestenbaum et al. 2025).

Although prognostication in neuropalliative care is still often difficult, it is necessary for specialist spiritual care providers to know about possible disease trajectories and crucial points where there might be a special need for a sensitive listener (e.g. after communication of a brain tumor diagnosis, diagnosis of a recurrent tumor, etc.) (Philip et al. 2020; Jones et al. 2021b). This might be especially important for adequate spiritual care planning and legacy work.

### Challenges in communication

Unique problems for clinical assessment of spiritual needs can occur in neuro-oncology and neurodegenerative patients. Individuals with cognitive impairment may lose the ability to articulate their spiritual needs, making non-verbal cues, life history, and contextual insight vital (Britt et al. 2022; Völz et al. 2024). Pathologies such as Parkinson’s Disease and Alzheimer’s dementia gradually impair verbal communication, making it harder for the individual to process information, express thoughts, and understand others. As the condition progresses, people often lose insight into their altered state and struggle to see things from another’s perspective. In primary and metastatic brain tumors, communication can be affected by tumor localization and progression, leading frequently to neurocognitive decline, including aphasia (IJzerman-Korevaar et al. 2018). Frequently, rapid clinical deterioration prevents therapies such as speech therapy or the use of devices from significantly delaying the deterioration of verbal communication skills.

In contrast, in ALS, muscle weakness gradually impairs speech and swallowing while cognitive function is often preserved, making alternative communication methods, such as eye-tracking communication systems, essential as the disease advances. Whenever health-care professionals are confronted with those alternative communication methods, they are required to know how to assist in communication. Flexibility, creativity, availability of time, and patience are essential. In the event of long disease trajectories, ongoing screening and assessment should consider the cognitive abilities at each time of assessment.

As the identification of spiritual needs may become more difficult as disease progresses, it is beneficial to discuss sources of spiritual strength and spiritual habits early in the therapeutic relationship, so that baseline information about spirituality can be collected and recorded in the patient’s medical records. For health-care providers, addressing spirituality in care also requires continuous attentiveness to how the underlying neurological changes may reshape the patient’s beliefs and values.

### Cultural safety in spiritual care

It has been argued that the spiritual care in palliative care is conceived in a Westernized, monotheistic manner thus demonstrating an irrelevance to non-Western communities, and not recognizing that religious beliefs are only a part of a person’s spiritual beliefs (Lundberg et al. 2024). This statement certainly contains some truth and therefore we call for cultural safety. Cultural safety creates an environment in which individuals feel spiritually respected, culturally acknowledged, and emotionally protected. It necessitates moving beyond knowledge of cultural or religious “facts” to self-reflection on power, bias, and historical inequities. For individuals with migrant backgrounds or belonging to minority spiritual traditions, standardized spiritual care may fall short. Thus, institutions must prioritize language access, multi-faith chaplaincy services, and care strategies that are codesigned with people who face or have faced health-related suffering. Culturally safe spiritual care improves engagement, trust, and well-being. In contrast, applying a one-size-fits-all model may result in therapeutic misconceptions and a lack of trust in health-care providers’ capacities or even loss of faith in health care systems (Paal et al. 2020). Spirituality should be addressed mindfully in a sensitive and professional manner to avoid causing additional harm. This includes, among other things, a judgment-free attitude toward the religious or spiritual beliefs of the person being addressed, a reflective approach to one’s own attitude toward spirituality, and a conscious design of the framework, timing, and objectives of the conversation. When assessing spirituality in a research context, it is essential to clarify the objectives and scope of the screening at an early stage to avoid misunderstandings in the sense of therapeutic expectations. At the same time, a plan for possible follow-up measures should be in place if relevant needs are expressed during the screening. At the end of a spiritual assessment, a proposal for further spiritual support should be made, if necessary. A follow-up appointment may be indicated to clarify the potential interventional character of such support (Paal et al. 2017).

### Spiritual care across care settings

As noted above, trying to learn about a person’s spirituality through taking a spiritual history and continuous spiritual screening represents a spiritual care intervention in itself. However, many spiritual care interventions are available for a range of specific care settings.

#### Clinical inpatient care

The clinical inpatient care setting is characterized by the availability of multidisciplinary care teams, which makes good communication skills and organization in terms of shared responsibilities for the persons’ spiritual needs necessary (Best et al. 2022). Health-care professionals can play a vital role in identifying and alleviating spiritual distress (Best et al. 2024). Embedding spirituality into care planning helps individuals to articulate what matters most and to ensure it is considered. This includes aligning medical interventions with deeply held values, preparing for death, or creating a sense of continuity through legacy practices (Garon et al. 2024b). Integrating interventions such as life review, storytelling, or rituals into care processes can help individuals and their families navigate transitions with purpose and peace (Austin *et al.* 2025). These practices can also support caregivers in their grieving and meaning-making processes (Zheng et al. 2021). Advance care planning should also encompass spiritual dimensions alongside clinical preferences (Kwak et al. 2024).

**Figure 1.**
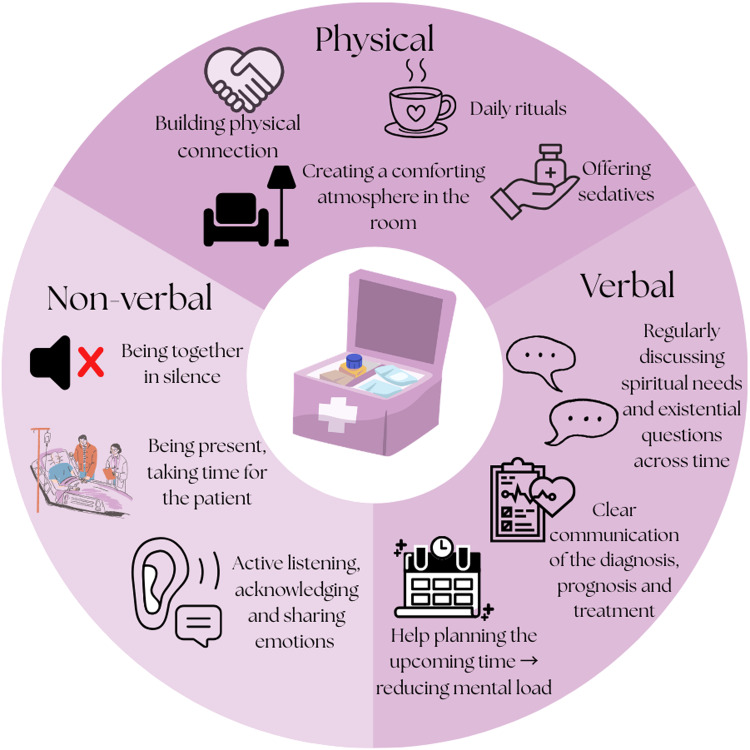
Spiritual toolbox for neuro-oncology. Adapted from Völz et al. (2025).

In any care setting, persons who are noncommunicative and/or have impaired consciousness tend to receive less spiritual care (Best et al. 2025). While basic comfort care has a spiritual dimension, more work is needed to identify effective interventions for spiritual distress. In addition to the difficulties in assessing spiritual needs, this is also due to limitations in appropriate intervention tools and the lack of practice, especially among spiritual care generalists. Therefore, health-care professionals should also be able to implement different approaches to spiritual care, such as physical and nonverbal measures (Figure 1; Völz et al. 2025).

#### Community care

In the context of outpatient care, outpatient clinics and long-term care facilities are primarily responsible for implementing spiritual care pathways and enabling documentation of spiritual needs and resources. This, along with the integration of spiritual screenings and care, helps spiritual care become a natural part of care plans. Currently, the lack of institutional policies and accountability mechanisms hinders integration of spiritual care (Galchutt and Peñate 2024).

Experience from a multiprofessional home care team for ALS patients showed that professional boundaries are often more fluid in home care than in institutional settings (Bublitz et al. 2025). This requires that team members define their roles with both clarity and flexibility and have the capacity to work autonomously, as a high degree of adaptability and responsiveness is needed to respond to the continually evolving needs encountered during home visits. Longitudinal engagement inherent in home care fosters deeper relationships and allows for more sustained attention to patients’ evolving spiritual needs. It is essential to recognize the patient’s home as a deeply personal and potentially sacred space, requiring professionals to establish their presence with humility and tact.

Whenever patients have complex care needs, diverse case management strategies must be considered, where people’s care is coordinated by a care coordinator, e.g. dementia care management (Reilly et al. 2015), Parkinson’s (Connor et al. 2019; Kluger et al. 2023), ALS (Soriani and Desnuelle 2017), and neuro-oncology (Bozzao et al. 2023). Persons working as case and care managers take on multidisciplinary perspectives and accompany the patients throughout their disease trajectory. As key workers in the organization of care, the case managers could be seen as gatekeepers to specialist spiritual care and, as such, should be able to conduct adequate spiritual assessment, so that the spiritual needs are identified and adequately addressed (Bozzao et al. 2023). Nurses who are responsible for the person-centered care coordination also have a primary role here (Grabenweger et al. 2025b).

Spiritual care interventions such as those used in the inpatient setting are also of value in community care. For long-term care, the introduction of ritual care would be helpful, especially for individuals with cognitive impairment (Butler et al. 2025). The advances in health-care digitalization show promising results in enhancing palliative care provision across patient settings (Maguraushe and Ndlovu 2024). In particular, telemedicine and video counseling have proven useful in the palliative care of patients with neurological diseases (Weck et al. 2019). These digital solutions contribute to improved care for patients in outpatient settings. However, there remains an evidence gap regarding digital spiritual care provision in general and within this specific patient cohort.

#### Specialist spiritual care chaplaincy

The multidisciplinary model of spiritual care, where the care team members are seen as spiritual care “generalists,” positions the chaplain as a crucial bridge between a patient’s worldview and the biomedical context, allowing them to provide personalized care through rituals, symbolic language, or simply a supportive presence (Bublitz et al. 2025).

Chaplains also have the time to administer more specialized interventions. The Hear My Voice program is an intervention developed for chaplains working with people with brain cancer or neurodegenerative diseases (Piderman et al. 2015). This program includes a spiritual legacy interview, which is summarized in a Spiritual Legacy Document in the form of a bound document. The development of a Legacy Document is also one of the aims of Dignity Therapy (Chochinov 2002; Fitchett et al. 2015), which proved to be an effective way to improve psychosocial well-being and quality of life for people with brain cancer (Piderman et al. 2015; Korman et al. 2021).

## Discussion

Based on our findings, we make a list of suggestions to be considered and implemented for clinical advancement, research, and education.

### Future directions for clinical advancement

To improve the practice of spiritual care, the following steps are recommended:

1. As spiritual care should be regarded as a shared responsibility, all members of interprofessional teams should be encouraged to initiate basic spiritual screening and make appropriate referrals, creating a culture where spiritual concerns can be openly discussed.
2. Include spiritual values in development of care plans and advance directives.
3. Promote codesigned rituals that reflect personal and cultural priorities.
4. Allocate time, training, and space for continuous team-based spiritual reflection and support. For example, nurses in ALS care face complex emotional and ethical challenges that call for strong institutional support and palliative care training. Enhancing palliative care integration from diagnosis, alongside targeted education and psychological support, is crucial to improving care quality and sustaining the well-being of both patients and nurses (Artioli et al. 2025).

### Future directions for research

The literature reflects a growing consensus on the importance of spiritual care but reveals persistent gaps.

#### Lack of validated screening tools

There is an urgent need for spiritual assessment instruments that are specifically tailored to patients with neurodegenerative and neuro-oncological diseases and validated in this population in order to gain further understanding of spiritual needs of people within neuropalliative settings (Grabenweger et al. 2025b). It has been proven that spiritual assessment tools in patients with neuro-oncological conditions are often not validated or are inappropriate due to their complexity and length (Liu et al. 2018; Grabenweger et al. 2025b).

In view of the communication challenges in neurological disease, such tools need to be developed and/or validated. Narrative and observational methods, complemented by interdisciplinary input, are needed to ensure spiritual concerns are not overlooked. Clinical guidance should emphasize the integration of open-ended, compassionate inquiry into standard assessments.

#### Lack of longitudinal research

Evidence demonstrates that spirituality is a lived and dynamic practice; thus, it should not be approached as a one-time intervention in clinical practice or research. Regular practices, like rituals, are impactful precisely because people with cognitive impairment can recognize the ritual character. Just as pharmacologic therapies require titration and adaptation, spiritual care requires continuity, flexibility, and presence. When spiritual interventions are approached as isolated events, especially for those unable to engage in dialogue, they may fail to meet the evolving needs of the person or, worse, provoke further alienation, frustration, or a sense of abandonment. This highlights an urgent need for the development and testing of embodied, sensory, and ritual-based interventions, such as touch, soundscapes, symbolic gestures, or aesthetic environments that do not rely on verbal exchange but can still evoke connection, belonging, and even affect sleep patterns, soothe restlessness, or lower heart rate.

Little is known about spiritual caregiving in long-term care facilities, often the location of those suffering from neurological disease (Murray and Pastrana 2024). Longitudinal studies of spiritual well-being and evaluation of spiritual care interventions are needed (Gonçalves et al. 2023).

#### Lack of effective interventions

Despite growing recognition of the importance of spiritual care in palliative settings, there remains a striking absence of well-evaluated, noninvasive, and nonverbal interventions designed to address spiritual distress, particularly among individuals with severe cognitive decline, aphasia, anarthria, or exhaustion. Current interventions that aim to alleviate existential suffering rely heavily on high-level verbal communication, narrative memory, and reflective capacity (Austin et al. 2025). While these methods have shown promise, they are inaccessible to many living with neuro-oncological or neurodegenerative conditions who are unable to engage verbally.

### Future directions for education

1. Surveys focusing on health-care professionals’ abilities to provide spiritual care (Völz et al. 2024; Bublitz et al. 2024b; Grabenweger et al. 2025b) point toward the need to focus on spiritual care competencies in education across health care sectors (Mason et al. 2020; Gatsios et al. 2021; Hökkä et al. 2024). All health-care professionals’ curricula should include core competencies in spiritual care, such as existential communication, cultural safety, and reflective practice modules.
2. The increasing prevalence of online tools for spiritual care education for health-care professionals (Fleenor 2022; Best et al. 2023; Grabenweger et al. 2025a) creates the possibility of greater expansion and improvement of spiritual care training (Jones et al. 2021a; Grabenweger et al. 2025a).
3. Cultural humility and trauma-informed approaches when teaching spirituality, religion, and health should guide all educational interventions (Paal 2024).
4. Interprofessional models should emphasize shared responsibility, while simulation-based learning and clinical supervision can help translate theory into practice (Best et al. 2020). Participants of an international neuropalliative course discussed the need for providing emotional support and building relationships to enhance the spiritual component of care as essential nonmedical treatment options (Bublitz et al. 2024b).
5. A review to identify how to overcome the education and practice gap (Brandstötter et al. 2022) demonstrated that organizations play a crucial role in enabling sustainable implementation of spiritual care by creating structures that integrate it into everyday practice. Collaboration with chaplains ensures access to professional expertise and helps embed spiritual care into interdisciplinary care models.
6. Providing diverse learning methods with an emphasis on practical application allows staff to develop skills and confidence in addressing spiritual needs in real-life situations. Identifying role models and mentors within the team fosters a culture of support and ongoing reflection. Creating adequate time, nurturing relationships, and maintaining a work environment that values holistic care are essential for sustaining spiritual care in practice.
7. Finally, developing clear training models and protocols ensures consistency, aligns spiritual with organizational standards, and supports its long-term integration into health-care delivery. Hence, to implement the spiritual care, institutions must establish referral pathways, protocols for documentation, and continuous opportunities for professionals to reflect on their values and emotional labor.

## Conclusion

On behalf of the EAPC Neuropalliative Care, Spiritual Care, and Education and Training Reference groups, we would like to emphasize that spiritual care is not a luxury or an optional extra; it is a fundamental aspect of palliative care. In view of its documented benefits for patients and their families, it is an ethical obligation and a practical necessity. For individuals navigating the profound disruptions of neuro-oncological and neurodegenerative illness, spiritual care upholds dignity, connection, and meaning. Institutions, clinicians, educators, researchers, and policymakers must collectively advance this agenda. By embedding spiritual care into clinical systems and educational models, we honor the full humanity of those we serve and accompany them through some of life’s most complex transitions.
